# Pin1 Exacerbates Non-Alcoholic Fatty Liver Disease by Enhancing Its Activity through Binding to ACC1

**DOI:** 10.3390/ijms25115822

**Published:** 2024-05-27

**Authors:** Yiyi Jin, Zhaoshui Shangguan, Jiao Pang, Yuwen Chen, Suijin Lin, Hekun Liu

**Affiliations:** Fujian Key Laboratory of Translational Research in Cancer and Neurodegenerative Diseases, The School of Basic Medical Sciences, Fujian Medical University, No. 1, Xuefu North Road, Fuzhou 350122, China; jinyiyi668@163.com (Y.J.); shangguan6471@163.com (Z.S.); pj_tian1314@163.com (J.P.); chenyuwen@fjmu.edu.cn (Y.C.); linsuijin@163.com (S.L.)

**Keywords:** Pin1, non-alcoholic fatty liver disease (NAFLD), AMPK, ACC1, phosphorylation

## Abstract

Non-alcoholic fatty liver disease (NAFLD) is a clinicopathological syndrome characterized by diffuse hepatocellular steatosis due to fatty deposits in hepatocytes, excluding alcohol and other known liver injury factors. However, there are no specific drugs for the clinical treatment of NAFLD. Therefore, research on the pathogenesis of NAFLD at the cellular and molecular levels is a promising approach to finding therapeutic targets and developing targeted drugs for NAFLD. Pin1 is highly expressed during adipogenesis and contributes to adipose differentiation, but its specific mechanism of action in NAFLD is unclear. In this study, we investigated the role of Pin1 in promoting the development of NAFLD and its potential mechanisms in vitro and in vivo. First, Pin1 was verified in the NAFLD model in vitro using MCD diet-fed mice by Western Blot, RT-qPCR and immunohistochemistry (IHC) assays. In the in vitro study, we used the oleic acid (OA) stimulation-induced lipid accumulation model and examined the lipid accumulation in each group of cells by oil red O staining as well as BODIPY staining. The results showed that knockdown of Pin1 inhibited lipid accumulation in hepatocytes in an in vitro lipid accumulation model and improved lipid indices and liver injury levels. Moreover, in vivo, WT and Pin1-KO mice were fed a methionine-choline deficient (MCD) diet for 4 weeks to induce the NAFLD model. The effects of Pin1 on lipid accumulation, hepatic fibrosis, and oxidative stress were evaluated by biochemical analysis, glucose and insulin tolerance tests, histological analysis, IHC, RT-qPCR and Western blot assays. The results indicate that Pin1 knockdown significantly alleviated hepatic steatosis, fibrosis and inflammation in MCD-induced NAFLD mice, improved glucose tolerance and alleviated insulin resistance in mice. Further studies showed that the AMPK/ACC1 signalling pathway might take part in the process by which Pin1 regulates NAFLD, as evidenced by the inhibition of the AMPK/ACC1 pathway. In addition, immunofluorescence (IF), coimmunoprecipitation (Co-IP) and GST pull-down experiments also showed that Pin1 interacts directly with ACC1 and inhibits ACC1 phosphorylation levels. Our study suggests that Pin1 promotes NAFLD progression by inhibiting the activation of the AMPK/ACC1 signalling pathway, and it is possible that this effect is achieved by Pin1 interacting with ACC1 and inhibiting the phosphorylation of ACC1.

## 1. Introduction

Non-alcoholic fatty liver disease (NAFLD) is a chronic liver disease that is increasing in incidence worldwide [1]. It is caused by ectopic fat accumulation in the liver, mainly owing to excessive fat uptake from foods, enhanced hepatic de novo lipid synthesis, and reduced fatty acid beta-oxidation [2]. NAFLD has a wide range of clinical pathologies. It represents a spectrum of liver disorders ranging from simple steatosis to non-alcoholic steatohepatitis (NASH), cirrhosis, and even hepatocellular carcinoma (HCC) [3,4,5]. However, the molecular pathogenesis of NASH remains largely elusive; thus, the treatment options for this disease remain limited.

To date, there is no FDA-approved treatment regimen for NAFLD patients. Lifestyle intervention involving reasonable diet and physical exercise is still the mainstay for NAFLD prevention and treatment. However, this strategy often has limited effects because of poor compliance [6,7,8]. The “double hit” hypothesis is the most widely accepted to explain the pathogenesis of NAFLD [9]. Insulin resistance (IR) and abnormal hepatocyte lipid accumulation represent the “first hit”, which in turn triggers oxidative stress, inflammation, apoptosis and fibrosis, the “second hit”, leading to the development of NASH. The “double hit” process destroys the dynamic balance between lipid synthesis and lipid consumption, resulting in excessive accumulation of liver fat and, in turn, leading to liver steatosis. Therefore, accelerating lipid consumption and/or inhibiting lipid synthesis is considered an effective solution to reducing hepatic lipid accumulation [10].

AMP-activated protein kinase (AMPK) is an essential cellular energy and nutrient sensor that is associated with the serine/threonine kinase family [11]. AMPK is a heterotrimeric complex consisting of a catalytic subunit (α) and two regulatory subunits (β and γ), which are generally expressed in eukaryotes. AMPK is activated by phosphorylation at Thr172 in the α subunit [12]. A great number of studies have verified that AMPK is a key regulatory factor of hepatic lipogenesis, making it a promising therapeutic target for the treatment of NAFLD [13]. The activation of AMPK may alleviate NAFLD by inhibiting liver de novo lipogenesis, increasing liver fatty acid oxidation, and augmenting adipose tissue mitochondrial function. Activation of AMPK plays an important role in controlling lipid metabolism by regulating the downstream acetyl-CoA carboxylase 1 (ACC1) and carnitine palmitoyltransferase I (CPT-1) pathways. Active AMPK inactivates ACC1, which inhibits the de novo synthesis of fatty acids and facilitates mitochondrial fatty acid β oxidation (FAO) mediated by CPT-1A [14,15,16]. On the other hand, sterol regulatory element-binding protein 1c (SREBP-1c) is the main regulator of fat acid synthase (FAS) and is also under the control of AMPK [17]. The activation of SREBP1 is one of the key factors of NAFLD. Decreasing the expression of SREBP1 can effectively reduce liver lipid deposition, and the activity could be inhibited by AMPK [18].

Pin1 is a unique peptidyl-prolyl cis-trans isomerase (PPIase) that can cis-trans isomerise the phosphorylated pSer/Thr-pro amino acid sequence in the substrate protein. Since its discovery, Pin1 has become well-established as a key factor in diverse cellular functions and in the pathogenesis of a range of diseases [19]. Several prior studies have focused on the development of malignancies and Alzheimer’s disease. Recent studies have shown that Pin1 is related to diabetes, NASH, obesity and other diseases.

In our study, we identified that Pin1 has a positive function in metabolic disorders in hepatocytes and the liver. Via in vitro experiments, we found that overexpression or knockout of Pin1 can exaggerate or ameliorate lipid accumulation and inflammation in hepatocytes, respectively. Consistently, further in vivo loss-of-function experiments demonstrated that Pin1 knockout had an inhibitory effect against hepatic steatosis, inflammation, insulin resistance, and fibrosis in NAFLD progression. Our findings introduce Pin1 as a novel therapeutic approach to suppressing the development of NAFLD.

## 2. Results

### 2.1. Pin1 Expression Is Upregulated in the NAFLD Model In Vitro and in MCD Diet-Fed Mice

**Figure 1:**
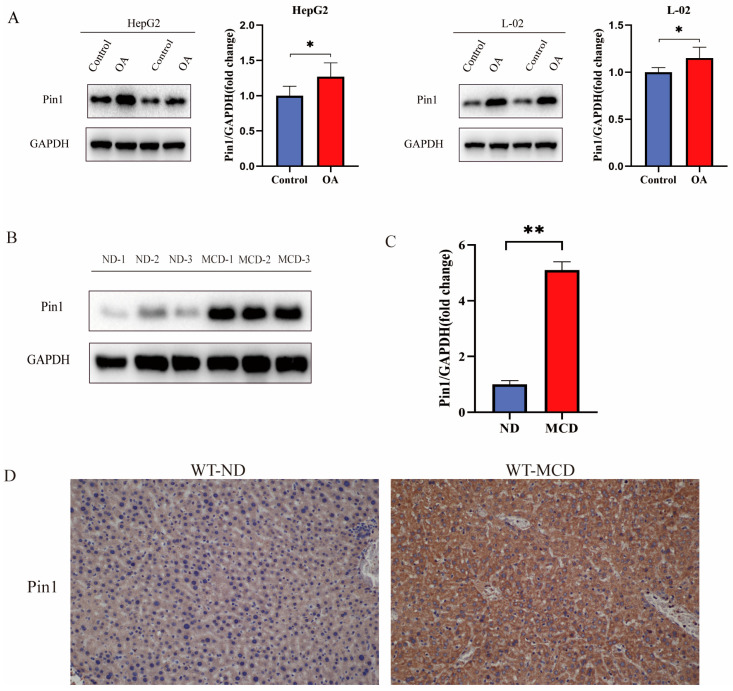
High expression of Pin1 in an in vitro lipid accumulation model and in vivo MCD-induced NAFLD model. (**A**) Protein and mRNA expression of Pin1 in the HepG2 cell model. (**B**) Protein and mRNA expression of Pin1 in the L-02 cell model. (**C**) Protein and mRNA expression of Pin1 in MCD-induced mouse liver tissue. (**D**) Immunohistochemical validation of Pin1 expression in MCD-induced mouse liver tissues (200×) (* *p* < 0.05, ** *p* < 0.01).

To determine the relevance of Pin1 in hepatic metabolism, we measured its expression in a cell model of NAFLD and in liver samples from MCD-induced mice. As shown in Figure 1A,B, the protein expression and mRNA levels of Pin1 were upregulated in the NAFLD group compared to the controls. Consistent with the findings in cells, Western blot analysis also showed that the hepatic levels of Pin1 were higher in mice fed an MCD diet than in mice fed a control diet; similarly, the mRNA levels of Pin1 in the MCD-fed group of mice were significantly higher than those in the normal diet control group (Figure 1C). Moreover, Pin1 protein was localized normally in hepatocytes from control mice but was significantly upregulated in the cytoplasm of steatotic hepatocytes from mice fed an MCD diet for 4 weeks (Figure 1D).

### 2.2. Pin1 Deficiency Inhibits Lipid Deposition In Vitro

**Figure 2:**
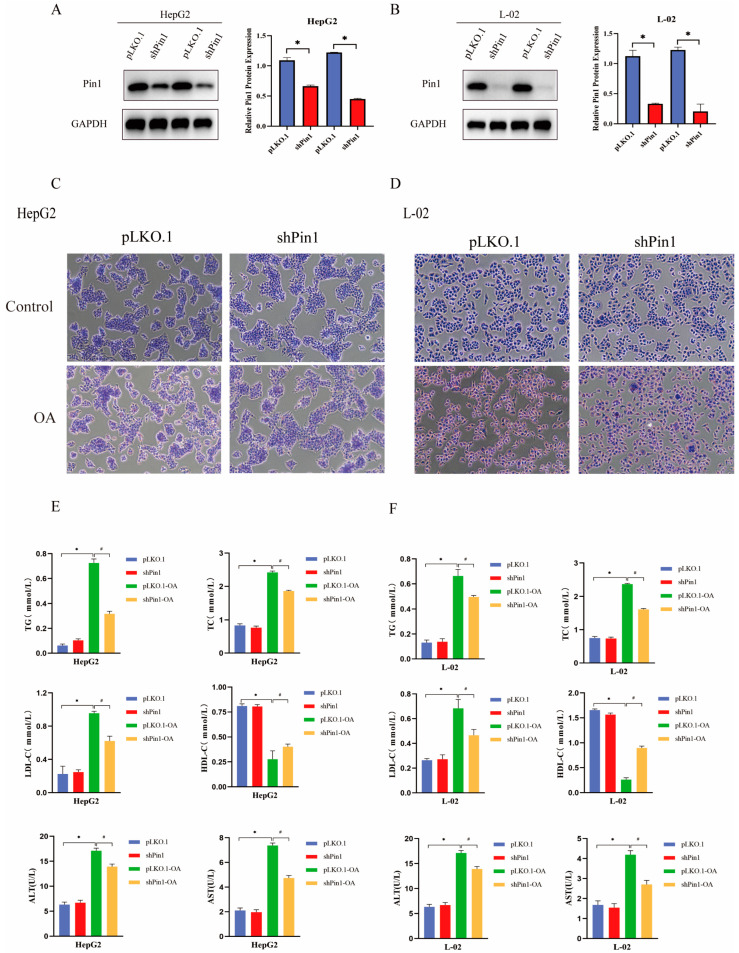
Knockdown of Pin1 inhibited lipid accumulation in an in vitro NAFLD model. (**A**) Western blot to verify the knockdown effect of Pin1 in HepG2 cells. (**B**) Western blot to verify the knockdown effect of Pin1 in L-02 cells (* *p* < 0.05). (**C**) Oil Red O staining in OA-induced HepG2 cells after Pin1 knockdown (100×). (**D**) Oil red O staining of OA-induced L-02 cells after Pin1 knockdown (100×). (**E**) Biochemical detection of changes in four levels of lipids and two indicators of liver injury in HepG2 cells after Pin1 knockdown. (**F**) Biochemical detection of changes in four levels of lipids and two indicators of liver function in L-02 cells after Pin1 knockdown (* indicates *p* < 0.05 compared with the pLKO.1 group; ^#^ indicates *p* < 0.05 compared with the pLKO.1-OA group, *p* < 0.05).

Before exploring the role of Pin1 in hepatic metabolic stimulation, we initially assessed the effect of Pin1 on in vitro lipid deposition and inflammation. We used lentiviral infection to construct stable transgenic HepG2 and L-02 cell lines. Pin1 protein expression was significantly decreased in all shPin1 cell lines (Figure 2A,B). Oil Red O results showed that there was a higher accumulation of neutral lipid droplets in the OA-induced cell model group, while lipid accumulation was slightly reduced in the HepG2 and L-02 cell models with Pin1 knockdown (Figure 2C,D). In NAFLD, the balance of lipid metabolism is often disturbed, mainly in the form of elevated TC, TG/TAG and LDL-c levels and decreased HDL-C levels. Thus, we collected the supernatants of cultured cells and measured the levels of two liver function items (ALT and AST) and four lipid items (TC, TG, HDL, and LDL). The results showed that in the in vitro-induced cell model, the levels of ALT and AST and TC, TG, LDL were significantly increased and the levels of HDL were decreased compared with the control group, while the levels of ALT and AST and TC, TG, LDL were decreased and the level of HDL was restored after knockdown of Pin1 (Figure 2E,F).

### 2.3. Pin1 Overexpression Exacerbates Lipid Deposition In Vitro

**Figure 3:**
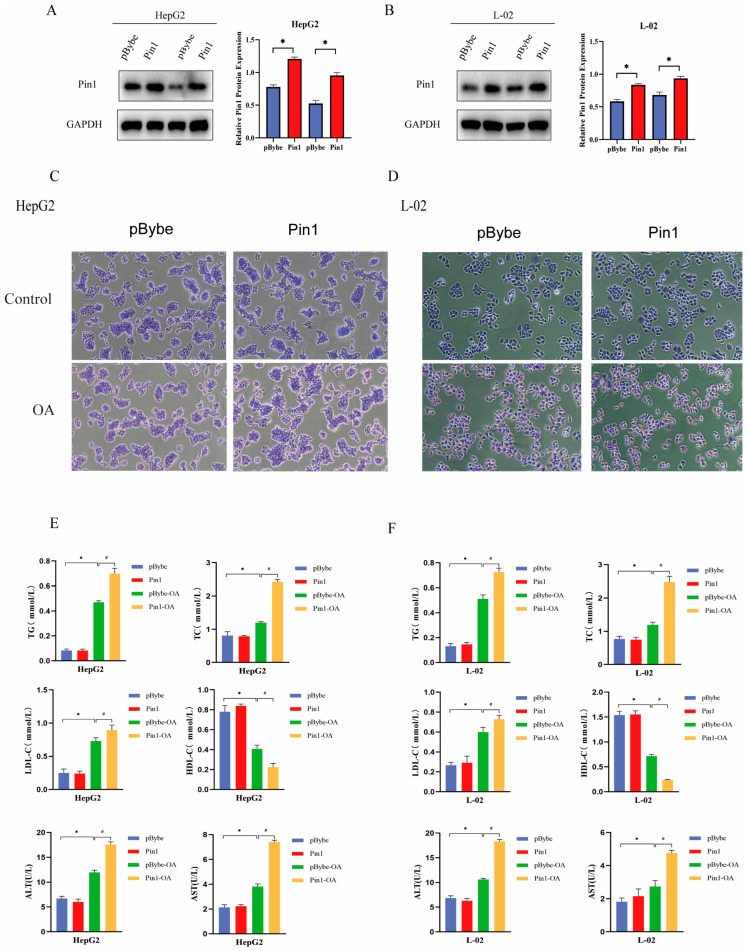
Pin1 overexpression exacerbates lipid accumulation in an in vitro cellular NAFLD model. (**A**) Western blot to verify the effect of Pin1 overexpression in HepG2 cells. (**B**) Western blot to verify the effect of Pin1 overexpression in L-02 cells (* *p* < 0.05). (**C**) Oil Red O staining of OA-induced HepG2 cells after Pin1 overexpression (100×). (**D**) Oil red O staining of OA-induced L-02 cells after Pin1 overexpression (100×). (**E**) Biochemical detection of changes in four levels of lipids and liver function in HepG2 cells after Pin1 overexpression. (**F**) Biochemical detection of changes in four levels of lipids and liver function in L-02 cells after Pin1 overexpression (* indicates *p* < 0.05 compared with the pLKO.1 group; ^#^ indicates *p* < 0.05 compared with the pLKO.1-OA group).

Similarly, we showed a significant increase in protein expression of Pin1 after overexpression of Pin1 in HepG2 and L-02 cells (Figure 3A,B), and oil red O results also showed that overexpression of Pin1 increased the accumulation of neutral lipids in cells (Figure 3C,D). Biochemical assays showed that overexpression of Pin1 significantly increased the levels of ALT, AST, TC, TG, and LDL and decreased the levels of HDL (Figure 3E,F), further indicating that Pin1 overexpression exacerbated lipid accumulation in NAFLD.

### 2.4. Pin1 Knockdown Stimulated the AMPK/ACC1 Signalling Pathway in NAFLD

**Figure 4:**
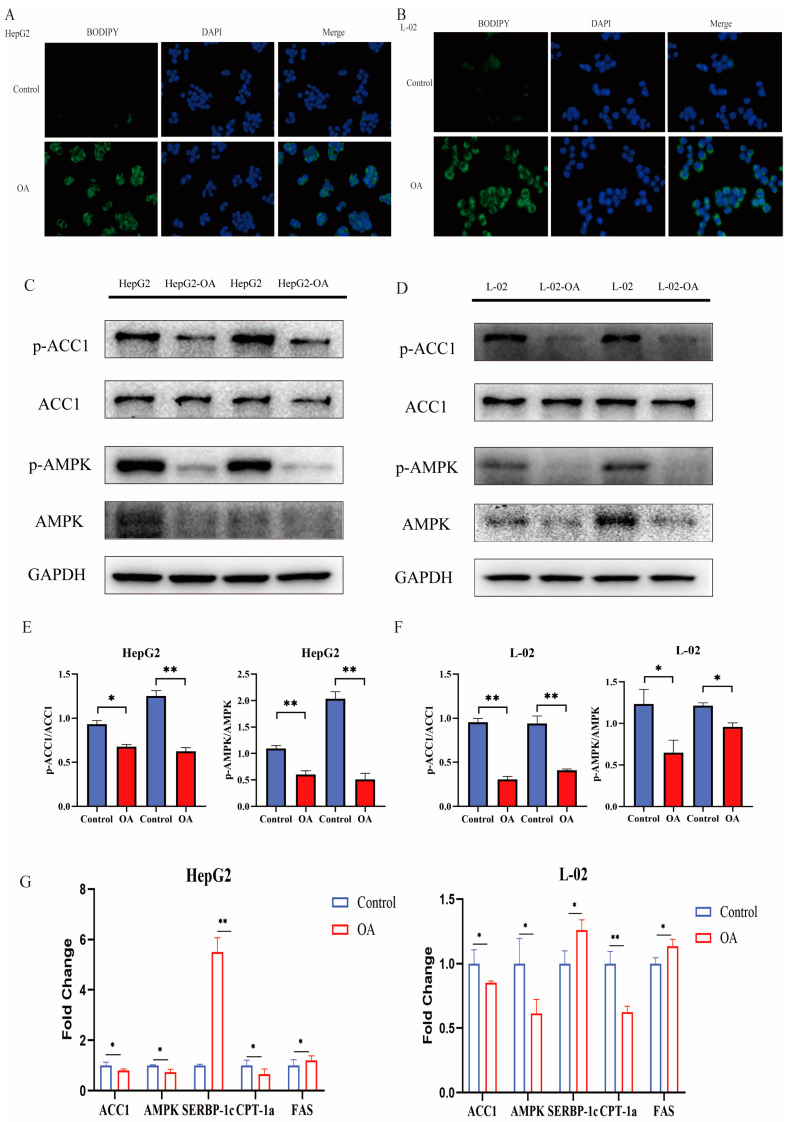
Pin1 inhibits the AMPK/ACC1 pathway in hepatocytes under metabolic stimulation. (**A**) BODIPY 493/503 staining of OA-induced HepG2 cells (200×). (**B**) BODIPY staining of OA-induced L-02 cells (200×). (**C**) Western blot to verify OA-induced changes in major molecules in the AMPK/ACC1 signalling pathway in HepG2 cells. (**D**) Western blot to verify OA-induced changes in major molecules in the AMPK/ACC1 signalling pathway in L-02 cells. (**E**) Quantification of p-ACC1/ACC1 and p-AMPK/AMPK protein levels in HepG2 cells normalized to GAPDH. (**F**) Quantification of p-ACC1/ACC1 and p-AMPK/AMPK protein levels in L-02 cells normalized to GAPDH (* *p* < 0.05, ** *p* < 0.01). (**G**) mRNA levels of genes in the AMPK/ACC1 pathway in the OA-induced cell model (* *p* < 0.05, ** *p* < 0.01).

**Figure 5:**
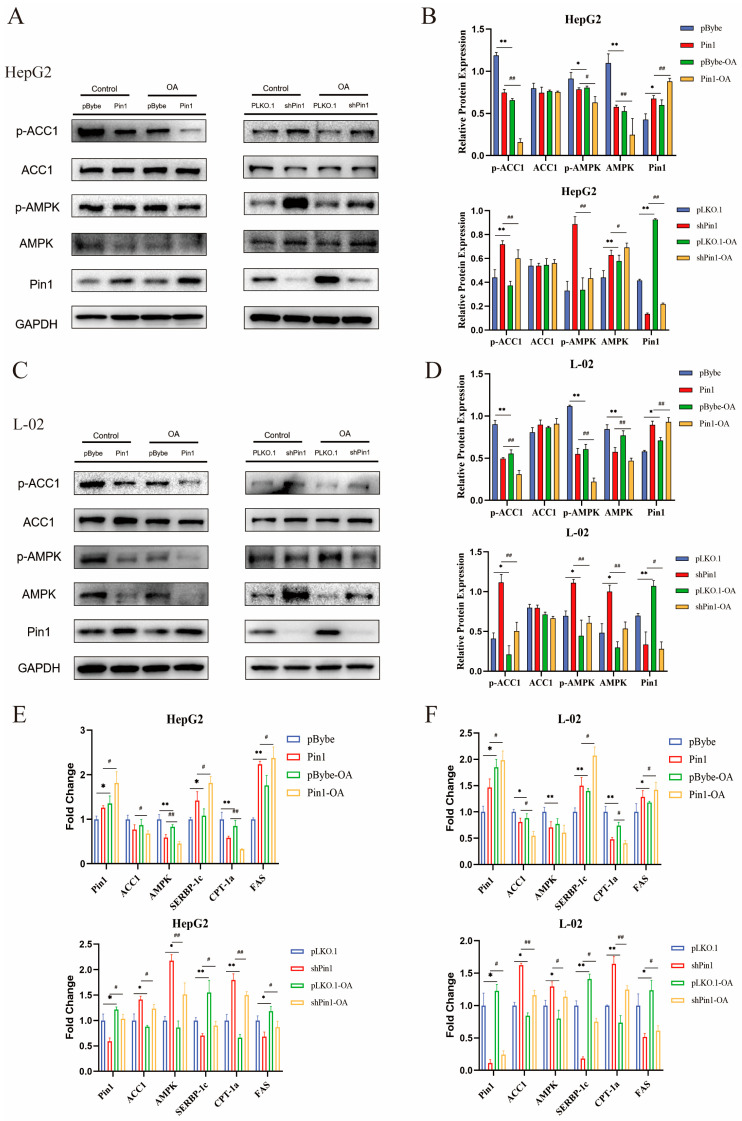
Pin1 inhibits the AMPK/ACC1 pathway in hepatocytes under metabolic stimulation. (**A**–**D**) Inhibition and activation of AMPK/ACC1 signalling pathway protein expression in HepG2 and L-02 cells after overexpression and knockdown of Pin1, respectively (* indicates *p* < 0.05, ** indicates *p* < 0.01; ^#^ indicates *p* < 0.05, ^##^ indicates *p* < 0.01). (**E**,**F**) mRNA levels of genes involved in the AMPK/ACC1 pathway in HepG2 and L-02 cells in OA-induced cell models after overexpression and knockdown of Pin1 (* indicates *p* < 0.05, ** indicates *p* < 0.01; ^#^ indicates *p* < 0.05, ^##^ indicates *p* < 0.01).

**Figure 6:**
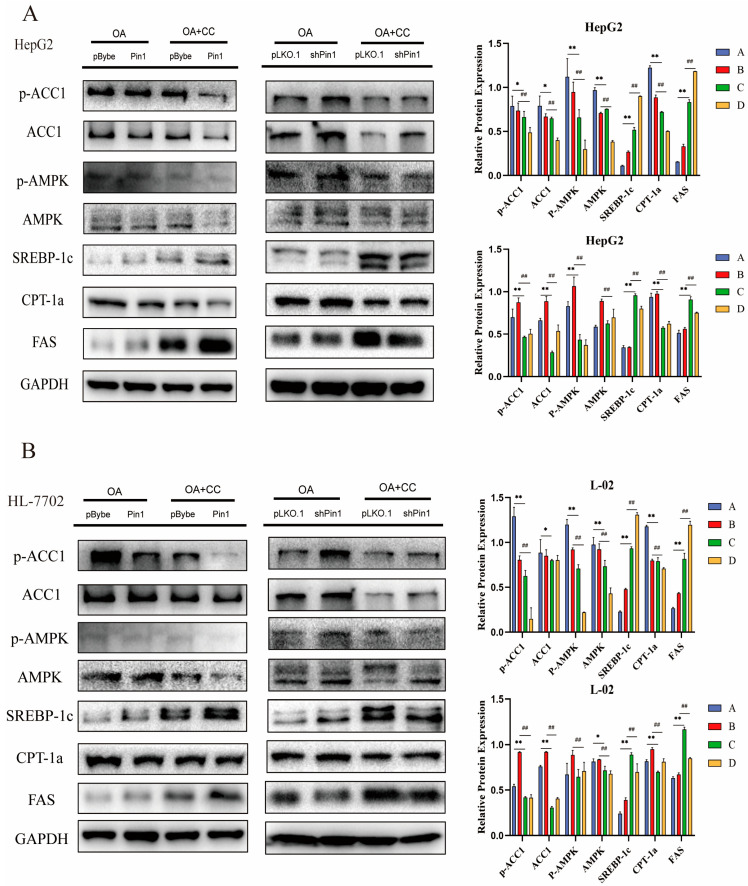
Pin1 inhibits the AMPK/ACC1 pathway in hepatocytes under metabolic stimulation. (**A**,**B**) Overexpression and knockdown of Pin1 in HepG2 and L-02 cells after the addition of the AMPK pathway inhibitor Compound C to validate AMPK/ACC1 signalling pathway protein expression and quantitative statistical plots, respectively. (* indicates *p* < 0.05, ** indicates *p* < 0.01; ^##^ indicates *p <* 0.01). Note: In the statistical graph of overexpressed cells, A indicates the pBybe-OA group, B indicates the Pin1-OA group, C indicates the pBybe-OA + CC group, and D indicates the Pin1-OA + CC group. In the statistical graph of knockdown cells, A indicates the pLKO.1-OA group, B indicates the shPin1-OA group, C indicates the pLKO.1-OA + CC group, and D indicates the shPin1-OA + CC group.

BODIPY 493/503 is a lipophilic fluorescent probe localized to polar lipids and can be used to label the neutral lipid content of cells, particularly those localized to lipid droplets. We used the BODIPY 493/503 fluorescent probe to further clarify the increased levels of neutral lipids in the cell model (Figure 4A,B). AMPK is a nutrient and energy receptor that maintains energy homeostasis and has significant therapeutic effects on obesity, insulin resistance, type 2 diabetes, cardiovascular disease and NAFLD by regulating multiple metabolic pathways [20]. ACC1 is phosphorylated and turned inactive by the activation of AMPK, quickly stopping the production of fatty acids [21]. Therefore, we conducted WB to evaluate the alterations in the AMPK/ACC1 pathway and discovered that total ACC1 expression was not significantly altered, whereas p-AMPK, AMPK, and p-ACC1 protein expression were all decreased in both cellular NAFLD models (Figure 4C,D). Additionally, Pin1 overexpression blocked the AMPK signalling pathway, which led to decreased ACC1 phosphorylation and enhanced fatty acid synthesis. In contrast, Pin1 suppression activated the AMPK pathway, phosphorylated and inactivated ACC1, and prevented the development of NAFLD (Figure 5A,C). We used the AMPK inhibitor Compound C (CC) to further validate the expression of related genes in the AMPK/ACC1 pathway based on overexpression and knockdown of Pin1. The results confirmed that Pin1 may promote the process of NAFLD by attenuating the activation of the AMPK signalling pathway (Figure 6A,B).

To determine the mRNA levels of related genes in the AMPK/ACC1 signalling pathway, we also performed an RT-qPCR assay (Figure 4G and Figure 5E,F). The experimental results are consistent with the WB results, further confirming our conclusions.

### 2.5. Pin1 Deletion Alleviates Non-Alcoholic Fatty Liver Disease and Hepatic Steatosis Induced by MCD

**Figure 7:**
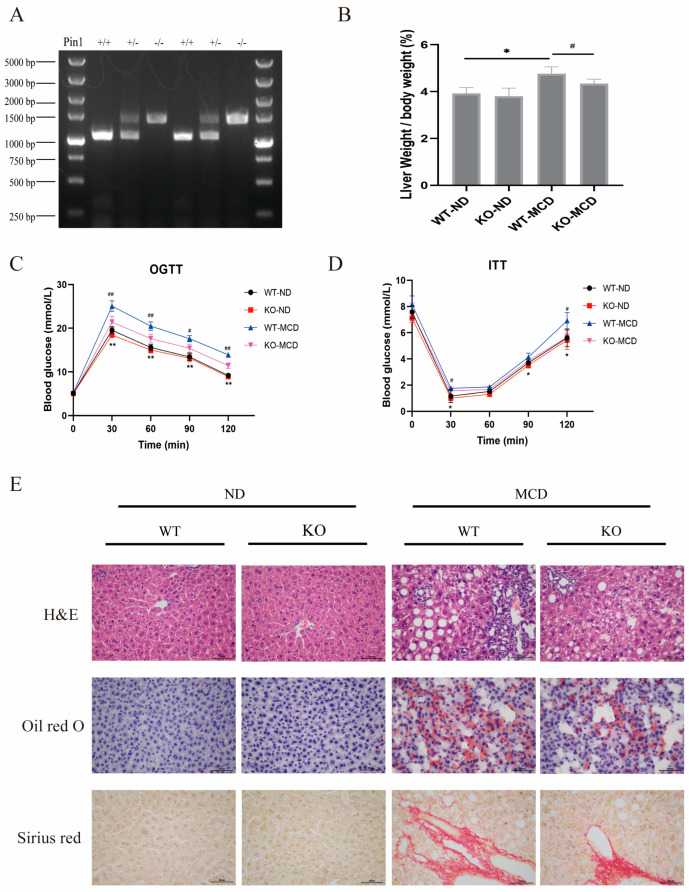
Pin1 deficiency alleviates MCD diet-induced NAFLD progression. (**A**) Identification of mouse genotypes. Pin1 wild-type (Pin1 +/+) mice had a band at 1000 bp, Pin1 heterozygous (Pin1 +/−) mice had two bands at 1000 bp and 1400 bp, and Pin1 knockout (Pin1 −/−) mice had a band at 1400 bp. (**B**) Pin1 knockout reduces the MCD diet-induced increase in liver index (* *p <* 0.05, ^#^*p <* 0.05). (**C**) Knockout of Pin1 improves glucose tolerance in mice after MCD diet feeding (** indicates *p <* 0.01 compared with the WT-ND group; ^#^ indicates *p <* 0.05 compared with the WT-MCD group; ^##^ indicates *p <* 0.01 compared with the WT-MCD group). (**D**) Knockout of Pin1 improves insulin sensitivity in mice after MCD diet feeding (* indicates compared with the WT-ND group; *p <* 0.05; ^#^ indicates *p <* 0.05 compared with the WT-MCD group). (**E**) Upper layer: HE staining to observe the effect of Pin1 knockout on histopathological changes in mouse liver tissues (400×); middle layer: Oil Red O staining to observe the effect of Pin1 knockout on lipid accumulation in mouse liver tissues (400×); lower layer: Sirius Scarlet staining to observe the effect of Pin1 knockout on the degree of fibrosis in mouse liver tissues (400×).

**Figure 8:**
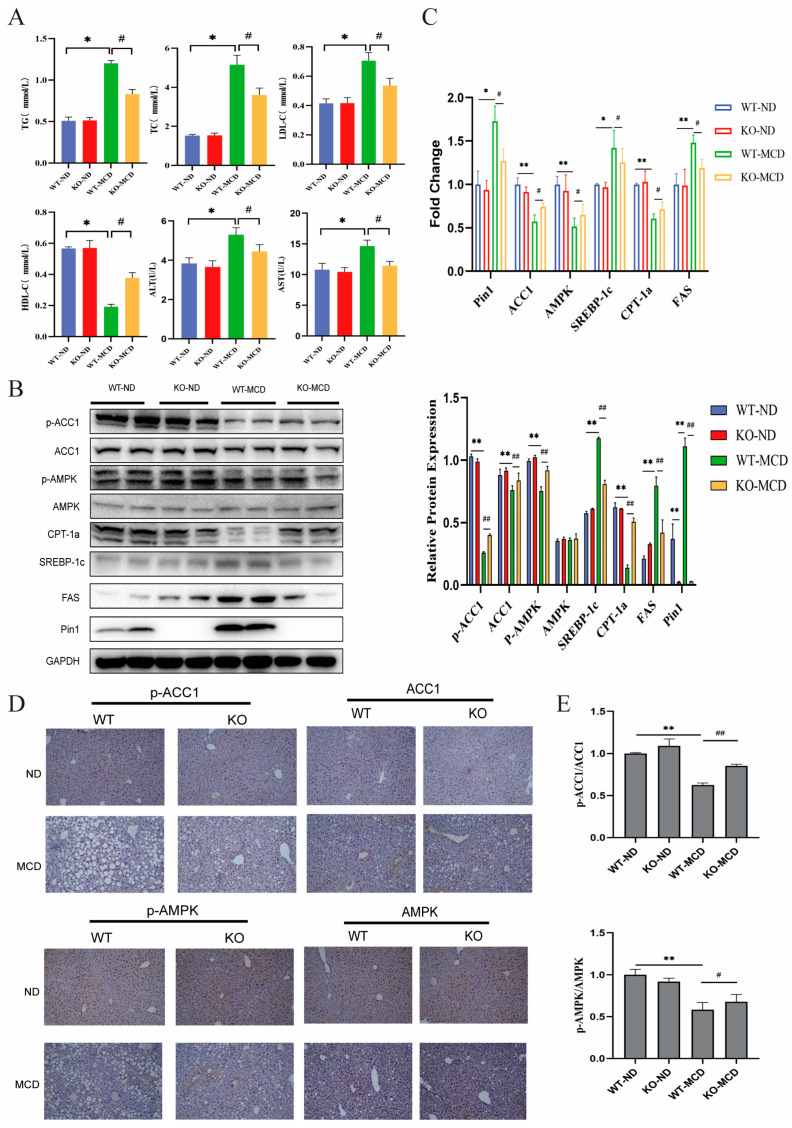
Pin1 deficiency alleviates MCD diet-induced NAFLD progression. (**A**) Knockout of Pin1 improved MCD-induced lipid levels and liver function impairment in mouse serum (* p < 0.05, ^#^*p <* 0.05). (**B**) Western blot detection and quantification of AMPK/ACC1 pathway protein expression in liver tissues of mice induced by MCD diet. (**C**) RT-qPCR was used to detect mRNA levels of related genes in AMPK/ACC1 pathway in liver tissue of mice induced by MCD diet (* indicates compared with WT-ND group, *p <* 0.05; ** indicates *p <* 0.01 compared with WT-ND group; ^#^ indicates *p <* 0.05 compared with WT-MCD group; ^##^ indicates *p <* 0.01 compared with WT-MCD group). (**D**) Immunohistochemical detection of p-ACC1, ACC1 and p-AMPK, AMPK protein expression levels in mouse liver tissues (200×). (**E**) Immunohistochemical protein expression quantification plots (** indicates *p <* 0.01 compared with the WT-ND group; ^#^ indicates *p <* 0.05 compared with the WT-MCD group; ^##^ indicates *p <* 0.01 compared with the WT-MCD group).

To further validate the effect of Pin1 deficiency on NAFLD progression, we subjected Pin1-KO and Pin1-WT mice to an MCD diet for 4 weeks to establish the NAFLD model. First, the genotypes of the mice were confirmed with PCR (Figure 7A). After consuming the MCD diet, the body weights of Pin1-WT and KO mice were comparable, but Pin1-WT mice exhibited a higher liver weight-to-body weight (LW/BW) ratio than Pin1-KO mice (Figure 7B). As revealed by the OGTT and ITT assays, glucose intolerance and insulin resistance were enhanced in Pin1-KO mice relative to Pin1-WT mice (Figure 7C,D). A remarkable decrease in hepatic lipid accumulation was shown in Pin1-KO mice compared with Pin1-WT mice, as exhibited by TG, TC, LDL-C and HDL-C contents (Figure 8A), H&E and Oil Red O staining, and the expression of genes modulating lipid metabolism. In addition, the results of Picric Sirius Red (PSR) staining revealed that liver fibrosis was less severe in Pin1 deficiency (Figure 7E). Immunohistochemical (IHC) staining showed that the AMPK/ACC1 signalling pathway was activated in Pin1-KO mice (Figure 8D), consistent with the results of the Western blot assay, RT-qPCR assay (Figure 8B,C) and in vitro experiments. Notably, serum ALT and AST concentrations also decreased markedly in Pin1-KO mice.

### 2.6. Pin1 Interacts with ACC1 and Prevents ACC1 from Being Phosphorylated

**Figure 9:**
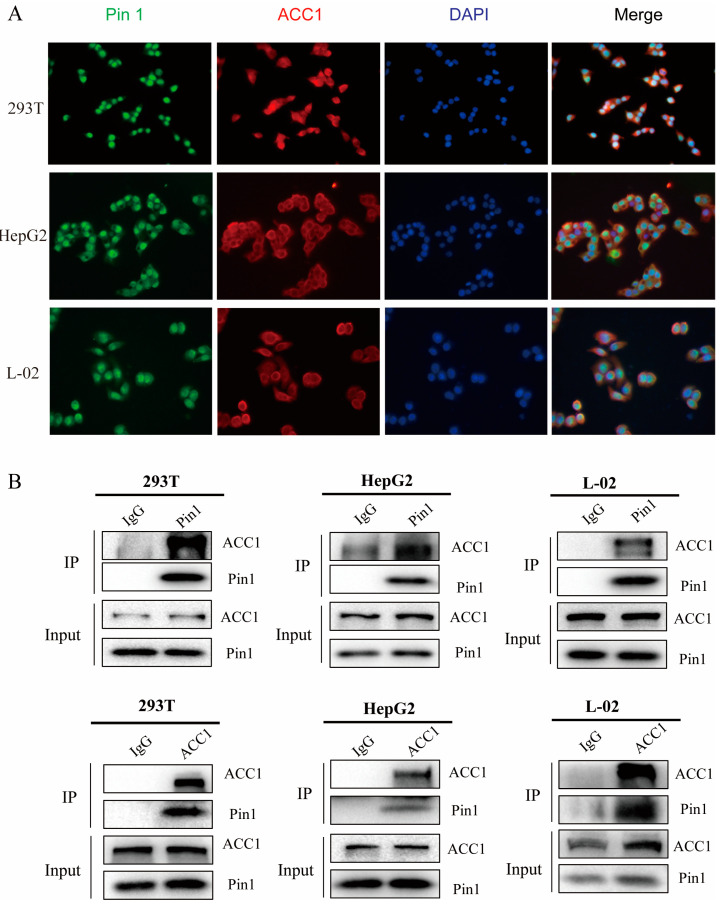
Pin1 interacts with ACC1 and blocks ACC1 phosphorylation. (**A**) Immunofluorescence assay results show the colocalization of Pin1 with ACC1 in cells (200×). (**B**) Co-IP detection of the interaction between Pin1 and ACC1 in cells.

**Figure 10:**
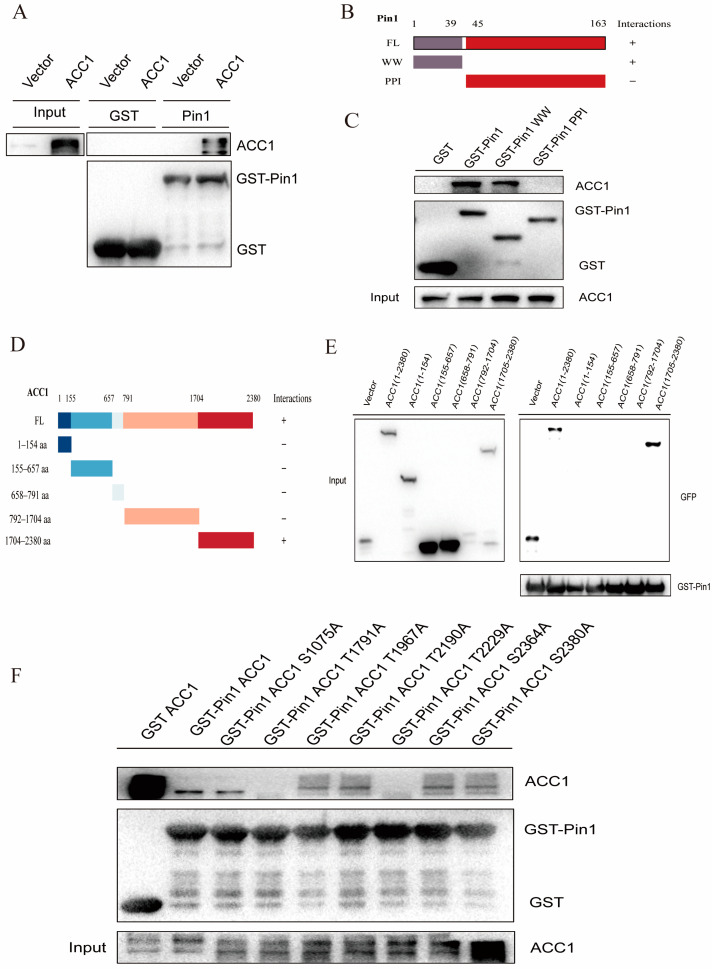
Pin1 interacts with ACC1 and blocks ACC1 phosphorylation. (**A**) GST pull-down assay detected the direct interaction between Pin1 and ACC1. (**B**) Schematic representation of the Pin1 WW and PPI structural domains. (**C**) GST-pull down detection of full-length ACC1 and Pin1 and Pin1 WW and PPI structural domain interactions. (**D**) Schematic representation of the 5 truncated bodies of ACC1. (**E**) GST pull-down detection of Pin1 and ACC1 full-length and 5 truncated body interactions. (**F**) The 1705-2380 domain containing T1791A and T2229A mutant ACC1 cannot bind to GST-Pin1 at all.

We explored how Pin1 and ACC1 interact in hepatocytes to modulate the downstream signalling that causes the progression of NAFLD. In 293T, HepG2, and L-02 cells, we utilized IF screening to reveal the colocalization of Pin1 and ACC1 (Figure 9A). Co-IP assays demonstrated that Pin1 could interact with exogenous and endogenous ACC1 (Figure 9B), which was also supported by the GST pull-down experiment (Figure 10A). Based on these findings, we used a variety of truncated segments of Pin1 and ACC1 to clarify the specific binding domain between the two proteins (Figure 10B,D). We discovered that the WW domain of Pin1 was essential for binding with ACC1 but not the PPI domain and that the 1705-2832 domain of ACC1 interacted with Pin1 but not the other four constructs (Figure 10C,E).

Given the direct interaction between the 1705-2380 structural domain of Pin1 and ACC1, to further identify specific binding sites, Thr or Ser in the seven Ser/Thr- pro motifs in the 1705-2380 structural domain region were replaced with Ala, and these GFP-tagged structures were overexpressed in HEK-293T cells and subjected to GST pull-down experiments. We found that both T1791A and T2229A mutants were completely unable to bind to GST-Pin1 (Figure 10F), suggesting that these two Ser/ThrPro motifs containing the 1705-2380 structural domain of ACC1 are essential for binding to Pin1.

## 3. Discussion

NAFLD is considered a precursor to metabolic syndrome and may further constitute a high risk of metabolism-related diseases such as dyslipidaemia, diabetes mellitus and cardiovascular disease [22,23,24]. It has been documented that lipid deposition, insulin resistance and inflammation mutually contribute to NAFLD and lead to liver dysfunction [25,26]. Therefore, it is crucial to stop this vicious cycle. Currently, the number of patients with NAFLD is increasing due to the prevalence of obesity, insulin resistance and metabolic syndrome [27], whereas effective treatment strategies for NAFLD are limited to lifestyle changes and dietary intake control since drug-based approaches have not shown any significant effect on the disease [28]. In this study, we sought to identify a target for the treatment of NAFLD and showed that Pin1 is a key contributor to MCD-induced hepatic steatosis. In addition, we demonstrated that Pin1 deficiency attenuates lipid accumulation due to NAFLD. Finally, our study demonstrated that Pin1 exerts its effects by blocking phosphorylation of ACC1 and activation of AMPK signalling. These findings suggest that Pin1 may be a promising target for the treatment of NAFLD.

Pin1 has become a crucial component of numerous cellular processes as well as the pathophysiology of a number of disorders since its discovery. Previous studies have concentrated on the development of malignancies and Alzheimer’s disease. Pin1 has now been linked to a number of diseases, including diabetes, NASH, obesity, osteoporosis, and cardiac hypertrophy. This has provided a preliminary demonstration of the physiological and pathogenic role of Pin1 in the regulation of metabolism [19]. MCD and high-fat diets (HFD) have both been shown to significantly boost Pin1 expression in the liver of NASH model mice. The development of hepatic steatosis, inflammation, and fibrosis in NASH mice was dramatically prevented by Pin1 loss, which is significant since it indicates that Pin1 in the liver is necessary for the development of steatosis [29]. In line with previous studies, our findings further show that Pin1 is strongly expressed in oleic acid-induced cell models and in the livers of MCD-induced mice. Pin1 overexpression or knockdown also significantly promotes or inhibits lipid accumulation in cell lines with metabolic stimulation, indicating its role in maintaining lipid metabolism homeostasis. Overall, Pin1 plays a catalytic role in metabolic disease in the pathogenesis of NAFLD.

Although the phenotype of Pin1 in NAFLD is well established, its specific mechanism of action has not been fully elucidated. AMPK is a kinase involved in a wide range of metabolic regulatory processes and is a major cellular “energy receptor”. AMPK plays an important role in maintaining lipid metabolism homeostasis by controlling lipid synthesis through the inhibition of lipid synthesis-related transcription factors [30]. In the process of lipid metabolism, ACC is a key enzyme in FAS and the oxidative pathway [31]. ACC1 catalyses the carboxylation of acetyl-CoA to malonyl-CoA and facilitates the de novo synthesis of FA, while ACC2 promotes the ab initio synthesis of FA by inhibiting ACC2, which inhibits CPT-1 and inhibits FA β-oxidation in mitochondria [32]. AMPK inhibits FA synthesis and promotes FA oxidation by inactivating both ACC1 and ACC2 through phosphorylation [33]. On the other hand, SREBPs are important nuclear transcription factors that regulate lipid metabolism. Currently, SREBP-1c mainly regulates FA metabolism, SREBP-2 mainly modulates TC metabolism, and SREBP-1a controls both FA and TC metabolism. FAS and GPAT are two key enzymes in the de novo synthesis of FA and TG, respectively, and both are regulated by SREBP-1 [34]. It has been demonstrated that AMPK in the liver inhibits the transcription of SREBP-1c and SREBP-2 genes by directly phosphorylating Ser372 of SREBP-1c and the unrecognized site of SREBP-2 [35,36], thereby inhibiting the expression of downstream FA and TC synthases [37] and reducing lipid synthesis. Thus, AMPK inhibits lipid synthesis, promotes lipolysis and oxidation, and plays a key role in the energy and lipid metabolism of the body. In the present study, we verified the effect of Pin1 on the AMPK/ACC1 signalling pathway, which was inhibited in a cellular model, while the inhibition of the AMPK/ACC1 signalling pathway was significantly enhanced after overexpression of Pin1, and its downstream target genes received corresponding effects. After knocking down Pin1, Pin1 showed the opposite regulatory effect, suggesting that Pin1 may promote the progression of NAFLD and exacerbate liver injury by regulating the AMPK/ACC1 signalling pathway.

To further clarify the role of Pin1 in NAFLD, we constructed an NAFLD model in Pin1 knockout mice and confirmed that knockdown of Pin1 reduced liver steatosis and fibrosis, while immunohistochemical results showed that knockdown of Pin1 could activate the AMPK/ACC1 signalling pathway and regulate the changes in its downstream target genes, reducing the lipid accumulation in the liver caused by NAFLD.

**Figure 11:**
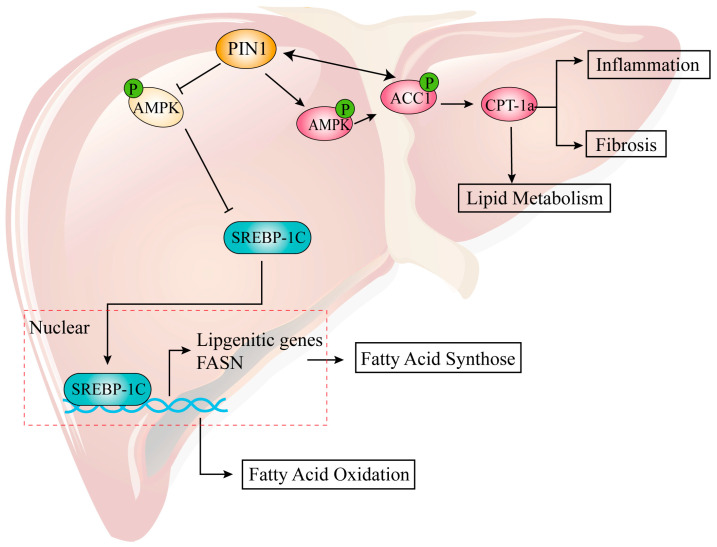
Diagram of the mechanism by which Pin1 regulates NAFLD. On the one hand, Pin1 promotes liver lipid aggregation, inflammation, and fibrosis by inhibiting AMPK mediated phosphorylation of ACC1 and downstream signaling pathway proteins, exacerbating non-alcoholic fatty liver disease; On the other hand, Pin1 regulates the activation of SREBP1, promotes the expression of fat synthase, aggravates liver lipid deposition, and promotes the process of NAFLD. During this process, Pin1 can enhance the activity of ACC1 by directly binding to block its phosphorylation, thereby accelerating the synthesis of fatty acids and promoting liver steatosis by acting on the AMPK/ACC1 pathway.

Elevated hepatic de novo lipogenesis (DNL) is a key factor in the development of NAFLD [38]. One of the core enzymes controlling DNL is acetyl-CoA carboxylase (ACC). ACC facilitates the conversion of acetyl-CoA to the metabolic intermediate malonyl-CoA. Malonyl-CoA is the first committed substrate of DNL and an inhibitor of fatty acid oxidation because of its xenobiotic inhibition of carnitine palmitoyltransferase-1. Thus, ACC is essential for controlling the flow of carbon intermediates between carbohydrate and fatty acid metabolism, and inhibition of ACC phosphorylation can inhibit adipogenesis [39]. Our study found that Pin1 can block ACC1 phosphorylation and enhance its activity by directly binding to ACC1, thus accelerating fatty acid synthesis and promoting hepatic steatosis by affecting the AMPK/ACC1 pathway, which may be an important mechanism by which Pin1 promotes the process of NAFLD (Figure 11). However, whether the binding of Pin1 and ACC1 in NAFLD affects other pathways and influences other pathogenesis remains unclear and is expected to be further explored.

In summary, this study identifies a role for Pin1 in promoting NAFLD progression. Pin1 exacerbates NAFLD by inhibiting AMPK-mediated inactivation of ACC1 and its downstream signalling pathways, promoting hepatic lipid aggregation, insulin resistance, and fibrosis. Mechanistically, the Pin1 WW domain directly interacts with the ACC1 1705-2832 domain, leading to the activation of ACC1, which in turn promotes the process of NAFLD. Our data provide mechanistic insights into the molecular regulation of NAFLD progression and suggest that Pin1 may be a valuable target for the treatment of NAFLD.

## 4. Materials and Methods

### 4.1. Chemicals and Reagents

Fetal bovine serum (FBS) was obtained from PAN Seratech (Aidenbach, Germany). Dulbecco’s modified Eagle’s medium (DMEM) was purchased from Thermo Fisher Scientific (Waltham, MA, USA). Oleic acid (OA) was obtained from Sigma–Aldrich Co. (St. Louis, MO, USA). Compound C was provided by MedChemExpress (Shanghai, China). The BODIPY 493/503 neutral lipid drop fluorescent probe was obtained from Shanghai Maokang (Shanghai, China). Trypsin, penicillin, streptomycin, 4′,6-diamidino-2-phenylindole (DAPI), Triton X-100, and the bicinchoninic acid (BCA) protein assay kit were purchased from Beyotime Institute of Biotechnology (Shanghai, China). The triglyceride assay kits and other biochemical assay kits were purchased from Nanjing Jiancheng Bioengineering Institute (Nanjing, China).

### 4.2. Cell Lines and Cell Culture

The hepatocellular carcinoma cell line HepG2, the immortalized normal human liver cell line HL-7702 (L-02) and human embryonic kidney-(HEK)-293T cells (purchased from the National Collection of Authenticated Cell Cultures, Shanghai, China) were cultured in DMEM supplemented with 10% FBS, 100 units/mL of penicillin, and 100 μg/mL of streptomycin under a humidified atmosphere of 95% air and 5% CO_2_ at 37 °C.

### 4.3. Lentiviral Transduction and Overexpression and Knockdown of Pin1 in Cell Lines

Recombinant lentiviral particles were produced in 293T cells by cotransfecting pLKO.1-puro and pBybe with helper plasmids pCMV-VSV-G, pRSV-Rev and pMDL. The virus-containing medium was harvested at 48 h after transfection and filtered using 0.22 µm filter. For infection, 500 µL virus medium was added to the HepG2 and L-02 cells with 1% polybrene, then the Pin1 overexpression and knockdown cells were selected with puromycin (2 µg/mL) for 72 h and verified by Western blot. Pin1-specific shRNA was designed as CCACCGTCACACAGTATTTAT.

### 4.4. Cell Model of NAFLD

HepG2 cells [40,41,42,43,44,45] and HL-7702 cells [46,47,48] are often used to induce fat accumulation and establish a non-alcoholic fatty liver disease model. HL-7702 cells (2 × 10^5^/well) and HepG2 cells (3 × 10^5^/well) were plated in 6-well plates and, when at 80% confluence, HL-7702 cells were incubated with 500 μM OA for 24 h, and HepG2 cells were incubated with 500 μM OA for 24 h to establish a steatosis cell model.

### 4.5. Observation of Lipid Accumulation

Cells were fixed in 4% paraformaldehyde for 20 min and rinsed with 60% freezing isopropanol for 5 min. Then, the cells were stained for 30 min at room temperature with freshly prepared oil red O working solution (Solarbio, Beijing, China), restained with haematoxylin and rinsed with flowing water. The cells were observed via inverted phase-contrast microscopy (Zeiss 2.3, Jena, Germany).

We subsequently performed BODIPY 493/503 staining, where cells were fixed in 4% paraformaldehyde for 10 min, washed twice with PBS, stained with BODIPY 493/503 2.5 μM for 30 min, washed with PBS and stained with DAPI for nuclei. Cells were observed via fluorescence microscopy (Zeiss 2.3, Jena, Germany).

### 4.6. Biochemical Measurements

At the end of the experiment, the eyes of mouse subjects were removed for blood collection, and approximately 1 mL of blood samples were collected. Subsequently, after sacrificing the mice, liver tissue was collected. Serum samples were obtained by centrifugation at 1790× *g* for 10 min at room temperature and later stored in microtubes at −80 °C. The levels of alanine aminotransferase (ALT), aspartate aminotransferase (AST), triglycerides (TGs), total cholesterol (TC), low-density lipoprotein cholesterol (LDL-C), and high-density lipoprotein cholesterol (HDL-C) were estimated using commercially available kits purchased from Nanjing Jiancheng Bioengineering Institute (Nanjing, China) in accordance with the manufacturer’s instructions.

After we cultured and induced the cells in groups, we collected cell supernatants and cell precipitates from each group and measured the TG, TC, LDL and HDL contents in the cells and the AST and ALT contents in the supernatant.

### 4.7. Western Blotting (WB) and Real-Time Quantitative PCR (RT–qPCR)

WB was performed using the standard method, as reported previously [49]. Anti-GAPDH antibody was used as a loading control to normalize the levels of other proteins. Total RNA in cells was extracted using TRIzol reagent (Invitrogen, Carlsbad, CA, USA) and reverse transcribed into complementary DNA with the PrimeScript Reverse Transcriptase kit (Takara, Dalian, China). RT-qPCR was performed on a 7500 Real-Time PCR system with a SYBR Real-Time PCR kit (Takara, Dalian, China). The relative gene expression level was calculated using the 2^−ΔΔCT^ method. All antibodies and primers are listed in Supplemental Tables S1 and S2.

### 4.8. Animals and Experimental Protocol

Experiments were performed in Pin1 knockout (KO) and wild-type (WT) mice. Pin1 KO mice have been described previously [50]. As Pin1 KO mice are infertile, Pin1 KO and WT littermates were obtained by mating with heterozygous mice. Mice were raised under a 12-h light/12-h dark cycle with food and water provided ad libitum. All experiments complied with the guidelines of the Animal Advisory Committee at Fujian Medical University and the US National Institutes of Health Guidelines for the Care and Use of Laboratory Animals. The animal study protocol was approved by the Animal Advisory Committee at Fujian Medical University (approval no. IACUC FJMU 2024-0004).

Eight-week-old male mice were used for the study experiments. At the beginning of the experiment, mice were randomly divided into the following 4 groups: WT and KO mice fed a normal diet (ND) were named WT-ND and KO-ND groups, respectively. Mice fed methionine-choline-deficient (MCD) diet, named WT-MCD group and KO-MCD group, respectively. Mice in each group were fed for 4 weeks [51]. During the experiment, body weights were measured once weekly. At the end of the experiments, blood was rapidly collected, all animals were anaesthetized and sacrificed, and the liver was excised for subsequent experiments.

### 4.9. Glucose and Insulin Tolerance Tests

Oral glucose tolerance tests (OGTTs) were performed in mice that were fed either a ND or MCD diet for 4 weeks. One week later, the same mice were used for insulin tolerance tests (ITTs). For OGTT, mice were fasted for 16 h. After measuring the baseline blood glucose level via a tail nick using a glucometer, 1.5 g/kg glucose was administered via intraperitoneal injection, and glucose levels were measured 0, 30, 60, 90 and 120 min after glucose injection. For ITT, 6 h fasted mice were injected intraperitoneally with recombinant human insulin at 0.5 U/kg, and their blood glucose concentrations were determined 0, 30, 60, 90 and 120 min after insulin injection.

### 4.10. Histological Analysis

Mouse liver sections were fixed in 4% formaldehyde and embedded in paraffin. After being cut into 4 μm sections, the tissues were stained with haematoxylin and eosin (H&E). Other parts of tissues were stained with Picric Sirius Red (PSR) and immunochemistry (IHC). The images were obtained under a microscope (Zeiss 2.3, Jena, Germany). In addition, hepatic cryosections were stained using an Oil Red O kit (Solarbio, Beijing, China) and counterstained using haematoxylin to observe lipid droplets.

### 4.11. Immunoprecipitation and GST Precipitation Assays

Immunoprecipitation assays were performed as previously described [47]. Briefly, cells were transfected for 36 h with the indicated plasmids, lysed in ice-cold immunoprecipitation buffer (50 mM Tris-HCl, pH 8.0, 150 mM NaCl, 1 mM EDTA, 1% Triton X-100 and 0.5% sodium deoxycholate) containing protease inhibitor cocktail tablets (#04693132001, Roche, Shanghai, China) and centrifuged at 14,000× *g* for 10 min. The cell lysates were then incubated with the indicated antibodies and protein A/G PLUS-Agarose (sc-2003, Santa Cruz, Shanghai, China) overnight at 4 °C followed by washing in a cold immunoprecipitation buffer. The immunocomplexes were collected and subjected to immunoblotting using the indicated primary antibodies and corresponding secondary antibodies.

GST-Pin1 fusion protein purification and GST pull-down assays. Rosetta (DE3) Escherichia coli was transformed with the GST-pin1 plasmid and then induced using 0.5 mM isopropyl-β-D-thiogalactopyranoside (IPTG) after the culture reached an optical density of 0.6–0.8 at 600 nm (OD600). E. coli extracts were prepared in PBS containing 0.5 mM MgCl_2_, 1 mM DTT, 1 mM PMSF and protease inhibitor cocktail tablets. BeyoGold™ GST-tag Purification Resin (P2262, Beyotime, Shanghai, China) was added, and the extracts were incubated at 4 °C for 2 h. The protein-loaded beads were then incubated at 4 °C for 4 h with cells that were prepared as described above for the IP assays. The isolated proteins were washed 4–6 times in the same buffer and subjected to Western blotting.

### 4.12. Immunofluorescence (IF) Experiments

After the cells were cultured, they were washed with PBS, fixed with 4% paraformaldehyde for 20 min, washed again, blocked with 3% BSA + 3/1000 Triton X-100 for 40 min, incubated with primary antibody overnight at 4 °C, added dropwise with the corresponding fluorochrome secondary antibody, and incubated at room temperature and protected from light for 2 h. After washing with PBS, the nuclei were restained with DAPI, incubated at room temperature and protected from light for 5 min, and finally photographed using fluorescence microscopy.

### 4.13. Statistical Analysis

Graphs were generated using GraphPad Prism 8.0 software, and data are presented as the mean ± SD. All in vitro experiments were performed at least three independent times. Statistical analyses were performed using SPSS 23.0. Comparisons between the two groups were performed using Student’s *t* test. One-way ANOVA followed by appropriate post hoc tests was used to compare more than two groups. *p* values < 0.05 were considered significant.

## Data Availability

All data generated or analyzed during this study are included in this article.

## Supplementary Materials

The following supporting information can be downloaded at: https://www.mdpi.com/article/10.3390/ijms25115822/s1.
