# Supplementary material for: Pin1 Exacerbates Non-Alcoholic Fatty Liver Disease by Enhancing Its Activity through Binding to ACC1
Source: Int J Mol Sci. 2024 May 27;25(11):5822. doi: 10.3390/ijms25115822 (PMC11171836; doi:10.3390/ijms25115822)
Supplement: Supplementary file 1 [file ijms-25-05822-s001.zip › ijms-3013962-supplementary.pdf]

## Supplemental Table S1:

**Table S1a.** Antibodies used for western blot or immunochemistry.

| Antibody | Company | Cat. No. | Species | Dilution |
|---|---|---|---|---|
| Anti-Pin1 antibody | Abcam | ab192036 | Rabbit | 1:1000 |
| AMPK Alpha 2 Polyclonal antibody | Proteintech | 18167-1-AP | Rabbit | WB: 1:1000<br>IHC: 1:200 |
| ACC1 Polyclonal antibody | Proteintech | 21923-1-AP | Rabbit | WB: 1:1000<br>IHC: 1:200 |
| Phospho-ACC1 (Ser79) Polyclonal antibody | Proteintech | 29119-1-AP | Rabbit | WB: 1:1000<br>IHC: 1:200 |
| Rabbit Anti-phospho-AMPK alpha 2 (Ser173) antibody | Bioss | bs-5575R | Rabbit | WB: 1:1000<br>IHC: 1:200 |
| FASN Polyclonal antibody | Proteintech | 10624-2-AP | Rabbit | 1:1000 |
| Anti-SREBP1antibody | Abcam | ab28481 | Rabbit | 1:1000 |
| Anti-CPT1A antibody | Abcam | ab234111 | Rabbit | 1:1000 |
| GFP-Tag(7G9) mAb | abmart | M20004M | Mouse | 1:1000 |
| GAPDH | TransGen Biotech | HC301-01 | Mouse | 1:5000 |

**Table S1b.** Primers used for qRT-PCR.

| Primer name | Primer sequence 5' – 3' |
|---|---|
| Pin1-Forward (homo) | GCAGCTCAGGCCGAGTGTA |
| Pin1-Reverse (homo) | TCCTTGGTCCGGGTGATCTT |
| ACC1- Forward (homo) | AGACTGTGGTGGTTGGTAGA |
| ACC1- Reverse (homo) | CTGCTGGATTACTTGGCTTCA |
| AMPK- Forward (homo) | CAACTATCGATCTTGCCAAAGG |
| AMPK- Reverse (homo) | AACAGGAGAAGAGTCAAGTGAG |
| CPT-1a-Forward (homo) | GGAGATTATCAACAAGCCAGACC |
| CPT-1a-Reverse (homo) | CACACCATAGCCGTCATCAG |

| | |
|---|---|
| SREBP1-Forward (homo) | CTTAGAGCGAGCACTGAACTG |
| SREBP1-Reverse (homo) | GGAACTGATGGAGAAGCTGTAG |
| FASN-Forward (homo) | CTCAGCCGCCATCTACAACA |
| FASN-Forward (homo) | GCCAGCGTCTTCCACACTAT |
| GAPDH-Forward (homo) | ACTAGGCGCTCACTGTTCTC |
| GAPDH-Reverse (homo) | GATCTCGCTCCTGGAAGATGG |
| Pin1-Forward (mus) | AAGATCACCAGGAGCAAGGA |
| Pin1-Reverse (mus) | AGAGCTGCAATCACTGAACTG |
| ACC1- Forward (mus) | ATGTTGAGACGCTGGTTTGTAG |
| ACC1- Reverse (mus) | TCTTCCTCTGTCAGTTGCTTCT |
| AMPK- Forward (mus) | GCAGTTGGATTATGAATGGAAGGT |
| AMPK- Reverse (mus) | CCGATCTCTGTGGAGTAGCA |
| CPT-1a-Forward (mus) | CGGCAGACCTATTTTGCACG |
| CPT-1a- Reverse (mus) | TAGATGCCTCAGGGTCCTCC |
| SREBP1-Forward (mus) | GGCTATTCCGTGAACATCTCCTA |
| SREBP1-Reverse (mus) | ATCCAAGGGCATCTGAGAACT |
| FASN-Forward (mus) | GGTGTGGAAGTTCGTCAGA |
| FASN-Forward (mus) | CAGTGTGCTCAGGTTCAGTT |
| GAPDH-Forward (mus) | TGGCAAAGTGGAGATTGTTG |
| GAPDH-Reverse (mus) | GGAAGATGGTGATGGGCTT |

**Supplemental Table S2:**

Table S2a. Quantitative analysis of steatosis (Oil Red O staining)

| Groups | Area of steatosis | Total area | Percentage of steatosis (%) |
|---|---|---|---|
| WT-ND group | 0 | 6291456 | 0.00% |
| KO-ND group | 0 | 6342153 | 0.00% |
| WT-HFD group | 2677918 | 5875793 | 45.58% |
| KO-HFD group | 1505347 | 5964732 | 25.24% |

Table S2b. Quantification of fibrosis (Picric Sirius Red staining)

| Groups | Area of fibrosis | Total area | Percentage of fibrosis (%) |
|---|---|---|---|
| WT-ND group | 0 | 6346982 | 0.00% |
| KO-ND group | 0 | 6313447 | 0.00% |
| WT-HFD group | 1511849 | 6049532 | 24.99% |
| KO-HFD group | 824673 | 6195847 | 13.31% |

Table S2c. NAS Scoring of NAFLD (H&E staining)

| Groups | Hepatocellular steatosis | Hepatolobular inflammation | Hepatocyte ballooning | Total score |
|---|---|---|---|---|
| WT-ND group | 0 | 0 | 0 | 0 |
| KO-ND group | 0 | 0 | 0 | 0 |
| WT-HFD group | 2 | 2 | 1 | 5 |
| KO-HFD group | 1 | 1 | 1 | 3 |
